# Hyperfrequent use and suicidal behavior in hospital psychiatric emergency services

**DOI:** 10.1192/j.eurpsy.2025.2370

**Published:** 2025-08-26

**Authors:** M. Valverde Barea, P. Vargas Melero, G. Ruiz Martinez, A. Jurado Arevalo, E. Perdiguero Sempere

**Affiliations:** 1 H. U. Jaén, Jaén, Spain

## Abstract

**Introduction:**

One of the great challenges for Mental Health Services is dealing with users who repeatedly use the facilities. This phenomenon, beyond representing an increase in the economic cost and in terms of human resources, generates high levels of frustration and dissatisfaction, both in professionals and in the consultants themselves. These users have been called “hyperfrequent users”. Users can frequent different services, including the Emergency service. The phenomenon of hyperfrequent use can lead to an inappropriate use of the Emergency services and gives rise to substantial costs for the health system, as well as a decrease in the efficiency of the service.

**Objectives:**

The objective of our work is to describe those factors associated with the hyper-frequent use of Mental Health Hospital Emergency Services by users who engage in suicidal behavior.

**Methods:**

A descriptive, observational study was carried out. The population included all users of the Hospital Emergency Department treated by the Mental Health Service in one year who consulted for suicidal behaviour (self-harming ideas, suicidal attempts or self-harm). Patients who consulted on 4 or more occasions in the hospital psychiatric emergency departments for consultations related to suicidal behaviour were considered as frequent users.

**Results:**

860 consultations were attended to, corresponding to 546 users who consulted in the psychiatric hospital emergency departments for suicidal behaviour (self-harming ideas, suicidal attempts or self-harm). Of these users, 314 consulted on more than one occasion. Taking as a frequent user >=4 consultations, we have 14 users in one year. Regarding sex, the female sex stands out 86% over the male sex 14%. One user is considered a great frequent user, attending on 17 occasions. The most frequent reasons for suicidal consultation among frequent users are consultations for self-harm ideation (33%) and self-harm attempts (60%) and self-harm (7%). Anxiety and alcohol consumption are the most frequent comorbid diagnoses among frequent users. By sex, self-harm behavior stands out in both women and men and self-harm is more frequent in women. Regarding discharge after assessment, referrals to a community mental health specialist stand out in 45% after consultation and 28% of frequent users required hospital admission after care for suicidal behavior.

**Conclusions:**

In our work, it is observed that the profile of frequent users with suicidal behavior is adult women who consult for self-harm attempts in their majority. These hyper-frequent users continue to demand attention from the health network devices, so knowing their needs would help to improve health care and use resources more efficiently and effectively for these users who engage in suicidal behavior.

**Disclosure of Interest:**

None Declared

